# Benchmarking machine learning robustness in Covid-19 genome sequence classification

**DOI:** 10.1038/s41598-023-31368-3

**Published:** 2023-03-13

**Authors:** Sarwan Ali, Bikram Sahoo, Alexander Zelikovsky, Pin-Yu Chen, Murray Patterson

**Affiliations:** 1 Department of Computer Science, Georgia State University, Atlanta, GA USA; 2 IBM T. J. Watson Research Center, Yorktown Heights, Yorktown, NY USA

**Keywords:** Computational biology and bioinformatics, Data mining

## Abstract

The rapid spread of the COVID-19 pandemic has resulted in an unprecedented amount of sequence data of the SARS-CoV-2 genome—millions of sequences and counting. This amount of data, while being orders of magnitude beyond the capacity of traditional approaches to understanding the diversity, dynamics, and evolution of viruses, is nonetheless a rich resource for machine learning (ML) approaches as alternatives for extracting such important information from these data. It is of hence utmost importance to design a framework for testing and benchmarking the robustness of these ML models. This paper makes the first effort (to our knowledge) to benchmark the robustness of ML models by simulating biological sequences with errors. In this paper, we introduce several ways to perturb SARS-CoV-2 genome sequences to mimic the error profiles of common sequencing platforms such as Illumina and PacBio. We show from experiments on a wide array of ML models that some simulation-based approaches with different perturbation budgets are more robust (and accurate) than others for specific embedding methods to certain noise simulations on the input sequences. Our benchmarking framework may assist researchers in properly assessing different ML models and help them understand the behavior of the SARS-CoV-2 virus or avoid possible future pandemics.

## Introduction

The novel Severe Acute Respiratory Syndrome Coronavirus 2 (SARS-CoV-2), a ribonucleic acid (RNA) coronavirus, was identified in January 2020^1^, which began the COVID-19 pandemic that is still ongoing today. With the help of sequencing technology and phylogenetic analysis, the scientific community disclosed that this novel coronavirus has 50% similarity with the Middle-Eastern Respiratory Syndrome Coronavirus (MERS-CoV), 79% sequencing similarity to Severe Acute Respiratory Syndrome Coronavirus (SARS-CoV)—also known simply as “SARS”—and more than 85% similarity with a coronavirus found in bats. Further studies confirmed that bats are the likely reservoir of these coronaviruses; however, the ecological separation of bats from humans indicates that some other organisms may have acted as intermediate hosts. Considering all scientific evidence, the International Committee on Taxonomy of Viruses named the novel RNA virus SARS-CoV-2^1–3^.

**Figure 1:**
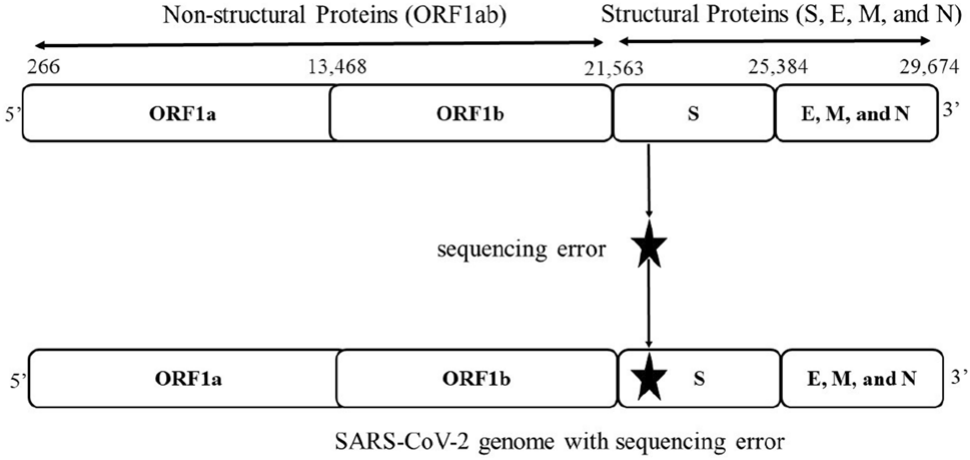
The SARS-CoV-2 genome codes for several proteins, including the surface, or spike (S) protein, where mutations happen disproportionately often^5,6^. Sequencing errors can bias the identification of a variant^7,8^. The above figure represents the incorporation of a sequencing error that appears as a mutation in the spike region of the SARS-CoV-2 virus. While such a sequence is part of a lineage in the phylogenetic tree, now it will be classed as being part of a different lineage because of the sequencing error. This figure is generated using “yEd Graph Editor” tool with Version 3.20.1 (https://www.yworks.com/products/yed).

RNA viruses generally introduce errors during replication, and the resulting mutations are incorporated into the viral genome after repeated replication within a single host, generating a heterogeneous population of viral quasi-species. However, SARS-CoV-2 has an excellent proofreading mechanism that encodes a nonstructural protein 14 (nsp14) allowing it to have a 10-fold lower mutation rate than typical RNA viruses. Epidemiologists estimate that SARS-CoV-2 undergoes 33 genomic mutations per year on average. Some of these mutations are advantageous, leading to more infectious variants of SARS-CoV-2 that continue to emerge^4^. Moreover, each major variant/lineage can be characterized or differentiated by a handful of mutations^5^. Hence, a sequencing error in the SARS-CoV-2 genome (see Fig. 1) may lead to a false variant/lineage and influence the current study of the SARS-CoV-2 virus^5,6^.

The diminishing cost of next-generation sequencing (NGS) technology has aided scientists from different parts of the world to generate large volumes of SARS-CoV-2 whole-genome sequencing (WGS) data. The Centers for Disease Control and Prevention (CDC) of the United States has also provided a wealth of information on resources, tools, and protocols for SARS-CoV-2 WGS data from different sequencing platforms such as Illumina, PacBio, and Ion Torrent. Finally, the Global Initiative on Sharing All Influenza Data (GISAID) hosts the largest SARS-CoV-2 genome sequencing dataset to date—the largest of any virus in history, with millions of sequences. This unprecedented amount of genomic data and easy availability allowed researchers to explore the molecular mechanism, genetic variability, evolutionary progress, and capability of development and spread of novel variants of the virus. On the other hand, this amount of data exceeds the capacity of the more traditional phylogenetic methods, such as Nextstrain^9^ or even the more recent IQTREE2^10^ by several orders of magnitude—a Big Data challenge. As a result, recent alternative approaches based on clustering and classification of sequences, e.g., to identify major variants have appeared in the literature^6,11–14^, with promising accuracy and scalability properties.

Many issues still remain, however, such as sequencing errors being mistaken for mutations in different analyses when studying the evolutionary and transmission patterns of SARS-CoV-2^8,15^, or other viruses. The incorporation of error in NGS sequences due to contamination in sample preparation, sequencing technology, or genome assembly methodology is another confounding factor. Generally, computational biologists filter those sequences with errors or mask those sequence fragments having errors. For example, each GISAID^16^ sequence is a consensus sequence from the intra-host viral population sampled from the patient, averaging out the minor variations which exist in this population. While such a consensus sequence is a good representative of this population, i.e., it is still precise enough to capture the SARS-CoV-2 variant harbored by the infected individual, it comes at the cost of losing this important information, such as these minor variations. Such minor variations, when given enough time to evolve, e.g., within an immunocompromised individual can become dominant—one of the theories behind the emergence of the Alpha variant^17^.

Many machine learning approaches towards clustering and classification of sequences^6,12,13^ have been operating under somewhat idealized conditions of virtually error-free consensus sequences, which may not be in certain settings. Moreover, some of these methods rely on a *k*-mer based feature vector representation—an approach that does not even rely on alignment of the sequences, which may not always be available in certain settings and can also introduce bias^18^. Such a framework should easily cope with errors as well—something machine learning approaches can do very naturally^19^. There are other methods in the literature for SARS-CoV-2 subtyping, such as Covidex^20^ and Nextclade^21^. Although these methods are proven to show higher predictive performance, it is not clear if they can be generalized to noisy sequence data. Hence, there is a great need for some way to reliably benchmark such methods for robustness to errors, which is what we carry out in this paper. Our main research question is the following:

*Given a perturbation budget, how robust are the existing classification models to noisy coronavirus inputs?*

In this paper, we extend our error testing procedure as a framework for benchmarking the performance of different ML methods in terms of classification accuracy and robustness to different types of simulated nucleotide sequences with errors. This involves using PBSIM and InSilicoSeq tools for simulating long reads and short reads-based nucleotide sequences with realistic error profiles. As the target label to perform classification, we use different lineages of coronavirus e.g. AY.103, AY.44, etc. In total, we extracted data for 41 unique lineages (class labels) from the GISAID database in January 2022.

We highlight the main contributions of this paper as follows:

- We propose several ways of introducing biologically meaningful errors into the SARS-CoV-2 genome sequences, which reflect the error profiles of modern NGS technologies such as Illumina and PacBio.
- Using different embedding methods from the biology domain such as PSSM Vector (based on the concept of position-specific scoring matrices) and Minimizer Vector (based on the concept of minimizers), we perform classification on original and errored sequences and report the performance using different evaluation metrics.
- We show that the alignment-free method for feature embedding, called *k*-mers Vector, which is based on the idea of *k*-mers, is better in terms of predictive performance when there is no error in the nucleotide sequences. This is likely due to the fact that it preserves nucleotide order information in more detail than the PSSM Vector or Minimizer Vector representations (at the expense of being less compact).
- We demonstrate that for the PBSIM (long reads) based errored sequences, the PSSM Vector embedding is more robust than the sliding window-based *k*-mers Vector or Minimizer Vector approaches, possibly because it captures more long-range information.
- We show that for the Illumina-based errored sequences, *k*-mers Vector and Minimizer Vector are able to show better performance than PSSM Vector, again because they likely preserve order information in higher detail.

The rest of the paper is organized as follows. In “Related work” section we discuss related work. The methods to generate the noisy examples are described in “Noise simulations creation” section. In “Feature embeddings generation” section, we discuss different embedding methods used to convert the sequences into fixed-length numerical representations. “Experimental setup” section contains the details regarding the experimental setup, dataset statistics, and data visualization. We report our results for accuracy and robustness in “Results and discussion” section. We described the limitations of our work in “Limitations” section. Finally, we conclude this paper in “Conclusion” section.

## Related work

### Robustness for noisy data in different domains

Assessing and benchmarking the robustness of ML or DL approaches by a series of noise simulations are popular in the image classification domain^22^, but there are others that are closer to the domain of molecular data. In^23^, the authors provide a series of realistic noise simulations to benchmark methods that predict chemical properties from atomistic simulations e.g., molecular conformation, reactions, and phase transitions. Even closer to the subject of our paper, the authors of^24^ show that methods, such as AlphaFold^25^ and RoseTTAFold^26^, which employs deep neural networks to predict protein conformation may not be robust: producing drastically different protein structures as a result of very small biologically meaningful perturbations in the protein sequence. Our approach is similar, albeit with a different goal of classification: namely, to explore how a small number of mutations (simulating the error introduced in certain types of NGS technologies) can affect the downstream classification of different machine learning and deep learning approaches.

### Kernel function based methods for sequence classification

Designing Kernel functions is a popular method for classification in the natural language processing (NLP) and bioinformatics domains for text and sequence classification, respectively^27–30^. These methods work by computing a kernel (distance) matrix based on the matches and mismatches between *k*-mers within sequences. The kernel matrix is used as input to traditional machine learning classifiers like support vector machine (SVM)^27–29^ for supervised analysis. There are two main problems in these methods, namely (1) kernel computation runtime and (2) storage of $n \times n$ dimensional matrix in memory when *n* (number of sequences) is large. Authors in^30^ proposed an efficient way of dimensionality reduction using information gain to speed up the kernel computation step. However, the space complexity issue still remains.

### Embedding generation methods for sequence classification

An alternative to kernel functions is to design fixed-length numerical embeddings, that can be used as input to machine learning classifiers for sequence classification. Authors in^6^ propose an embedding method for the classification of spike sequence data. However, their approach is not alignment-free nor scalable to larger datasets. Neural network-based methods, such as Wasserstein Distance Guided Representation Learning (WDGRL)^31^ and AutoEncoder^32^ have been proposed in the literature to obtain the embeddings for sequences given one-hot encoding-based vectors as input. An end-to-end deep learning model is also proposed in^33^ for genome data analysis. These neural network-based methods, however, take a lot of time to train and usually generalize poorly on test data. Another embedding generation method for gene sequences, called DMk, is proposed in^34^. However, the resultant embeddings are specifically designed for the clustering task, hence not applicable in our case since we are performing sequence classification.

### Bioinformatics tools

Some bioinformatics tools have been proposed in the literature for SARS-CoV-2 subtyping, such as Covidex^20^ and Nextclade^21^. These tools show higher predictive accuracy of biological sequences in general. However, they are not designed to deal with noisy data, hence generalize poorly when given errored sequences for testing, as we see in the results.

## Noise simulations creation

We use two types of approaches to generate noisy examples so that we can test the robustness of different machine-learning methods.

### PBSIM simulated data generation

PBSIM is developed to simulate Pacific Biosciences (PacBio) sequencing reads. Generally, the PacBio sequencer generates two types of reads: continuous long reads (CLR) and circular consensus sequencing short reads (CCS). The CLR reads have a high error rate, and CCS reads have a lower error rate. PBSIM can simulate both CLR and CCS reads with different approaches: sampling-based simulation and model-based simulation. In the sampling-based simulation, PBSIM considers the length and quality of a provided read set to simulate the reads. In the model-based simulation, PBSIM simulates the reads on the basis of an error model^35^.

To generate a sequence with errors (perturbed sequences), we take an original SARS-CoV-2 genomic sequence and simulate reads from it using the model-based approach with the default PacBio error model. These reads (containing errors) are then aligned to the original sequence, mutations are called, and then consensus sequences (with mutations, some of which are errors) are extracted. We control the amount of error (perturbation budget) in the reads by adjusting the depth of the reads (a parameter of PBSIM). More specifically, we generate a perturbed sequence for each of the 8220 different SARS-CoV-2 sequences from GISAID for reading depths 5, 10, 15, and 20.

### InSilicoSeq simulated data generation

The InSilicoSeq open-source tool simulates the reads from different short-read technologies such as Illumina. InSilicoSeq is a widely used tool, and several studies generate more realistic NGS data using this tool for planning new experiments and benchmarking purposes^36–40^. The tool can incorporate errors into the reads based on recent Illumina platform details (e.g., chemistry). InSilicoSeq supports substitution, insertion, and deletion errors and can model the PHRED score. The current release of the InSilicoSeq tool has a pre-built error model for HiSeq, MiSeq, and NovaSeq instruments. Moreover, InSilicoSeq has the option to generate the number of reads according to the user’s requirement^41^.

We generate a sequence with errors analogously to the above, this time controlling the error (perturbation budget) by adjusting this number of reads. We generate a sequence for the 8220 GISAID sequences mentioned above, with a number of reads 50,00, 10,000, 15,000, and 20,000.

#### Remark 1

Note that we selected PBSIM and InSilicoSeq to generate noisy examples because they are well-known methods from the literature. It is important to use these tools because the main challenge while generating noisy examples for biological sequences is to introduce the error in “*biologically meaningful way*” so that the biological structure of nucleotide sequences is not disturbed and the resulting sequences do not look synthetic (for example, we cannot introduce some random error in the sequences as it will disturb the structure of nucleotide sequences). This way, perturbed sequences highly resemble true biological sequences yet, at the same time, may fool a classifier.

## Feature embeddings generation

This section introduces different feature embedding methods used to convert the nucleotide sequence into a fixed-length representation.

### *k*-mers vector^14^

**Figure 2:**
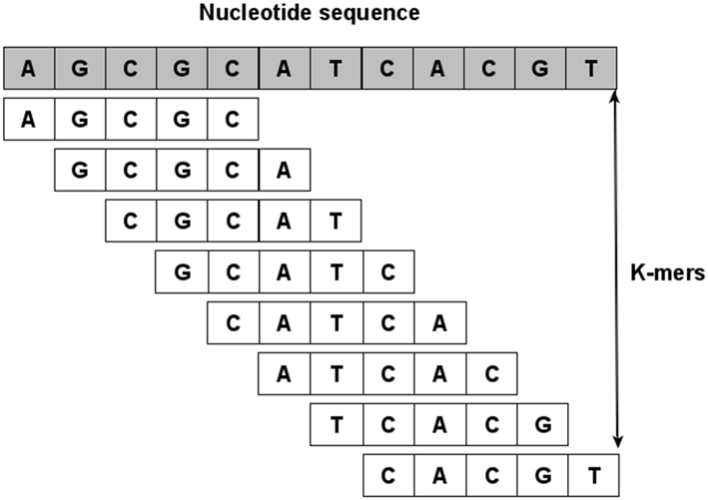
Example of different *k*-mers in a nucleotide sequence. This figure is generated using “yEd Graph Editor” tool with Version 3.20.1 (https://www.yworks.com/products/yed).

A popular approach to preserve the ordering of the sequential information, called Spike2Vec^14^, takes the sliding window-based substrings (called mers) of length *k* (also called *n*-gram). This *k*-mers-based representation helps to preserve the order of characters within the sequences^12^ (see Fig. 2 for an example of *k*-mers).

First, the *k*-mers are computed for each nucleotide sequence in this approach. Then a fixed length frequency vector is generated corresponding to each sequence, which contains the count of each *k*-mer in that sequence. One advantage of using *k*-mers based approach is that it is an *“alignment-free”* method unlike other popular baselines (e.g., one-hot encoding “OHE”^6,12^), which requires the sequences to be aligned. In one-hot encoding, each nucleotide is represented by a 0-1 binary vector of length 4 (because of 4 nucleotides in every sequence). Since unaligned sequences can have a different number of nucleotides, hence the resultant one-hot representation will also have variable length. Although we can use methods such as data padding to make these one-hot vectors have similar lengths, the pairwise distance information, however, is lost to a certain extent. Due to these issues, one-hot encoding requires aligned sequences as input. Note that sequence alignment is expensive and requires a reference sequence (genome)^42,43^. It may also introduce bias into the result^18^. The total number of *k*-mers in a given nucleotide sequence is $N - k + 1$, where *N* is the length of the sequence. The variable *k* is the user-defined parameter. In this paper, we take $k = 3$ (decided empirically using standard validation set approach^44^).

#### Frequency vector generation

After generating the *k*-mers, the next step is to generate the fixed-length numerical representation (frequency vector) for the set of *k*-mers in a nucleotide sequence. Suppose the set of nucleotides in the whole dataset is represented by the alphabet $\Sigma$ (A, C, G, and T). Now, the length of the frequency vector will be $\vert \Sigma \vert ^k$ (all possible combinations of *k*-mers in $\Sigma$ of length *k*). Note that this length is fixed for all sequences regardless of their sequence length. Hence, no matter the number of *k*-mers extracted from a given nucleotide sequence, since the length of the frequency vector is constant, this method can work on variable length sequences (hence exhibits the alignment-free property). In our dataset, since we have 4 unique nucleotides in any sequence, the length of the frequency vector in our case would be $4^3 = 64$ (when we take $k = 3$).

After getting the *k*-mers frequency vectors, authors in Spike2Vec apply random Fourier feature^45^ approach to reduce the dimensionality of the data. Since our dataset is comparatively smaller, we did not apply that method, hence we refer to this method as *k*-mers Vector rather than Spike2Vec in our paper.

### PSSM vector

The PSSM Vector embedding is based on the idea of the position-specific scoring matrix (PSSM), also called position weight matrix (PWM)^46,47^. For a given nucleotide sequence *s*, PSSM Vector designs the PWM. The PWM generation starts by first computing the *k*-mers (where $k = 3$, which is decided using standard validation set approach^44^) for *s*. For all the *k*-mers in *s*, a matrix of length $\vert \Sigma \vert \times k$ is generated, which includes the count of nucleotides at different positions within the *k*-mers. This matrix is also called the position frequency matrix (PFM). In the next step, column-wise probabilities are computed for PFM to get a new matrix called the position probability matrix (PPM). More formally, the PPM is computed as follows:

1 $$\begin{aligned} \frac{\text {Frequency of nucleotide}}{\text {No. of nucleotides in the column}}. \end{aligned}$$

To avoid having zero in the denominator, we add a small value of 0.01 (called Laplace value or pseudocount) during the probability computation. Finally, the PWM is computed from the PPM by taking the log-likelihood of each nucleotide $c \in \Sigma$ at a position *i*. More formally:

2 $$\begin{aligned} W_{c, i}=\log _{2} \frac{p(c, i)}{p(c)} \end{aligned}$$

where $p(c) = \frac{1}{4}$, which corresponds to the equal probability of occurrence for each nucleotide in the sequence. After generating the PWM, we flatten the matrix to generate a single vector, which we refer to as PSSM Vector.

### Minimizer vector

The Minimizer Vector feature embedding is based on the idea of minimizers^48^. The minimizer is a modified version of a *k*-mer and is used to represent a biological sequence in a more compact form.

#### Definition 1

(*Minimizers*) For a given *k*-mer, a minimizer (also called m-mer) is a substring of consecutive nucleotides of length *m* from the *k*-mer, which is lexicographically smallest one in both forward and backward order of the k-mer, where $m<k$ and is fixed.

The pseudocode to compute the minimizers is given in Algorithm 1. For a better understanding of pseudocode, we use the syntax of python code. To compute the minimizers, we take $k=9$ and $m=3$, which is decided empirically using standard validation set approach^44^. After computing the minimizers for a given nucleotide sequence, we follow the same method to generate the frequency vector-based representation as described in “Frequency vector generation” section. For reference, we denote this method as Minimizer Vector.
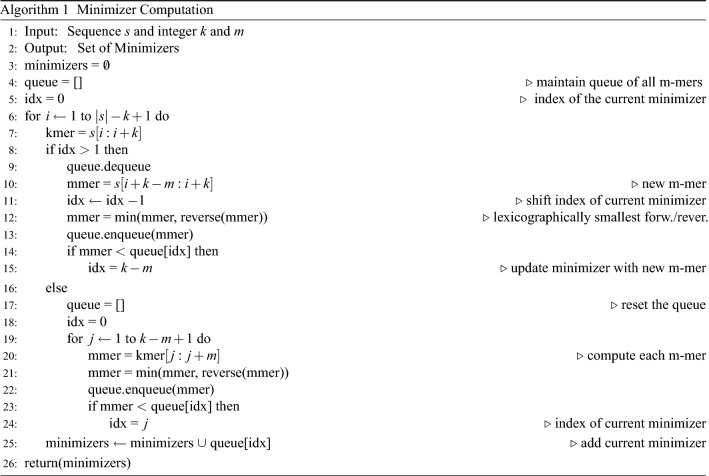


#### Remark 2

The goal of selecting these embedding methods is that they are alignment-free and also showed the best results in terms of predictive performance. Moreover, the *k*-mers, minimizers, and position weight matrix are one of the most common methods used in the bioinformatics domain for sequence analysis.

We also use the tools such as Covidex^20^ and Nextclade^21^ for classifying the lineages. These tools simply take the nucleotide sequences as input and give us the lineage name as the predicted value. No training is involved for these methods as they are “pre-trained” on a set of biological sequences.

## Experimental setup

All experiments are conducted using an Intel(R) Core i5 (11th generation) with a 2.40 GHz processor having windows 10 (64 bit) OS and 32 GB memory. The simulated and pre-processed data is available online (https://github.com/sarwanpasha/Adversarial_attack_on_biological_sequences). For classification purposes, we use the Support Vector Machine (SVM), Naive Bayes (NB), Multi-Layer Perceptron (MLP), K-Nearest Neighbors (KNN), Random Forest (RF), Logistic Regression (LR), and Decision Tree (DT).

To measure the performance of ML models, we apply the following two different strategies.

*Accuracy* In this case, we compute the average accuracy, precision, recall, F1 (weighted), F1 (Macro), and ROC-AUC for the whole (original) dataset (with respect to the lineages “class labels” reported in Table 1) without any errored sequence.

*Robustness* An important characteristic of the robustness of models is their ability to provide sensible outputs when input examples are not drawn from the training data^49,50^. Therefore, in this strategy, we only consider the noisy examples (set of errored sequences) for the test set (and non-errored sequences for the training set) and compute average accuracy, precision, recall, F1 (weighted), F1 (Macro), and ROC-AUC for the ML models.

### Dataset statistics

We used the full-length nucleotide sequences of coronavirus from a popular and publicly available database of SARS-CoV-2, GISAID (https://www.gisaid.org/). In order to collect the error-free sequences, we selected specific parameters while downloading the data from GISAID, such as full-length SARS-CoV-2 sequences, generated from high-coverage reads, ensuring that these sequences are virtually error-free. In total, we extracted 10, 000 nucleotide sequences from GISAID. After preprocessing (removing those sequences for which the lineage count was $<10$) we came up with 8220 sequences. We selected these nucleotide sequences along with their COVID-19 lineage information in January 2022.

The total number of unique lineages (class labels) in our dataset is 41. The dataset statistics for the prepossessed data are given in Table 1 (the first and the third column show the class labels, which are the lineages of SARS-CoV-2, while the second and fourth column shows the proportion of sequences corresponding to each lineage). Given this set of nucleotide sequences, our problem is to classify the lineages (class labels), and we do so by converting the sequences into fixed-length numerical vectors using different embedding methods. Our simulated dataset is available online for reproducibility (https://drive.google.com/drive/folders/1adtr8FImIYTqxM20wgInRqIZ8EJY4HVS?usp=sharing).

**Table 1:** Dataset statistics for different SARS-CoV-2 lineages in our data.

Lineage	No. of sequences	Lineage	No. of sequences
AY.103	2271	AY.121	40
AY.44	1416	AY.75	37
AY.100	717	AY.3.1	30
AY.3	710	AY.3.3	28
AY.25	585	AY.107	27
AY.25.1	382	AY.34.1	25
AY.39	248	AY.46.6	21
AY.119	242	AY.98.1	20
B.1.617.2	175	AY.13	19
AY.20	130	AY.116.1	18
AY.26	107	AY.126	17
AY.4	100	AY.114	15
AY.117	94	AY.125	14
AY.113	94	AY.34	14
AY.118	86	AY.46.1	14
AY.43	85	AY.92	13
AY.122	84	AY.98	12
BA.1	79	AY.46.4	12
AY.119.2	74	AY.127	12
AY.47	73	AY.111	10
AY.39.1	70	_	_

After preprocessing, the total number of sequences (and corresponding lineages) is 8220.

### Comparison with DL models

It is well known from the literature that deep learning (DL) methods (and other ML classifiers) do not work efficiently as compared to simple tree-based methods in the case of tabular data^51–53^. However, to validate that concept, we use a pre-trained model called SeqVec^54^, and other DL methods such as LSTM^55^, GRU^56^, and CNN^57^ for sequence classification on original data. Following is the detail regarding different DL models:

#### LSTM

The LSTM architecture consists of an embedding layer (of length 500), an LSTM layer with 200 memory units, a LeakyReLU layer with alpha = 0.05, an LSTM layer again with 200 memory units followed by another LeakyReLU layer, a dropout with value 0.2, a Dense layer of dimensions 500 followed by LeakyReLU layer, and finally an output layer and a sigmoid activation function. We use the ADAM optimizer in this architecture.

#### GRU

The GRU architecture consists of an embedding layer (size of embedding is 500), a GRU layer with 200 memory units, a LeakyReLU layer with alpha = 0.05 followed by a Dropout layer with value 0.2, and finally, a dense output layer and a sigmoid activation function. We also use the ADAM optimizer in the GRU architecture.

#### CNN

Similarly, the CNN architecture comprises an embedding layer (size of embedding is 500), a 1-D convolution layer (Conv1D) with 128 filters and a kernel size of 5, a LeakyReLU layer with alpha = 0.05, a batch normalization layer, a 1-D convolution layer (Conv1D) again with 128 filters and a kernel size of 5, a LeakyReLU layer with alpha = 0.05 followed by batch normalization, a max pooling layer with pool size equals 2, a dense layer of 500 dimensions followed by a LeakyReLU layer with alpha = 0.05, and finally an output dense layer with a sigmoid activation function. For optimization, we use the ADAM optimizer.

#### SeqVec^54^

The SeqVec is a pre-trained language model for biological sequences that use Embeddings from Language Models (ELMO)^58^ for its training. Given biological sequences as input, we fine-tune the model based on our input data and it outputs the embeddings for the sequences. The resultant embeddings are context-based and used as input to classical machine learning classifiers for supervised analysis.

### Data visualization

**Figure 3:**
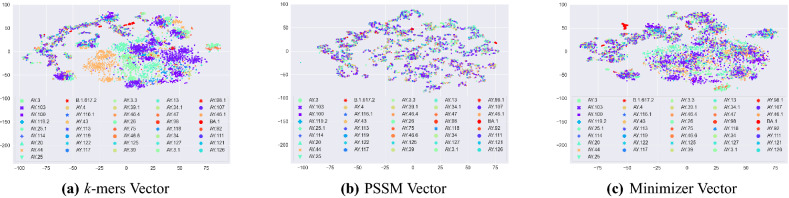
t-SNE plot for different embedding methods. This figure is generated using “Matplotlib” library in python with version 3.3.2 (https://matplotlib.org/).

To visualize if there is any (natural) clustering in our data, we generated a 2D representation of the feature embeddings using the t-distributed stochastic neighbor embedding (t-SNE) approach^59^. The main advantage of t-SNE is that it preserves the pair-wise distance between vectors in 2 dimensions. The t-SNE plot for different coronavirus variants is given in Fig. 3a–c for *k*-mers Vector, PSSM Vector, and Minimizer Vector, respectively. For the *k*-mers Vector-based t-SNE plot, we can observe that some of the variants (e.g., AY.103) are grouped more clearly than the other variants. PSSM Vector, however, maintains smaller groups of variants as compared to *k*-mers Vector. The structure of Minimizer Vector-based t-SNE is more similar to *k*-mers Vector. However, it grouped some other variants (e.g., AY.96.1) more clearly as compared to *k*-mers Vector. In general, we can observe that all embedding methods preserve the overall structure of the data.

## Results and discussion

In this section, we report the performance of ML models using two metrics, namely accuracy and robustness. For the accuracy metric, we report classification results for different ML models on the original data (without noisy examples). For the robustness results, we trained the classifiers on the original sequences (without any error) and tested their performance on the errored sequences.

### Accuracy results

To evaluate the performance of original nucleotide sequences (non-errored sequences), we split the sequences into (random) 70–30% training and testing set and perform classification on different embedding methods. To demonstrate that our results are not dependent on the specific random splits of data, we ran the experiments 5 times and reported average results.

#### Remark 3

Note that we also performed a 5 fold cross-validation, and the results were not very different from the average results of 5 random runs.

The accuracy results for different embeddings and ML models are shown in Table 2. We can observe that the SVM classifiers with *k*-mers Vector-based embedding outperform other embeddings and ML models for all but one evaluation metric. In terms of runtime, since the length of vectors for PSSM Vector is smaller than the other embedding methods, its training runtime for the NB classifier is the smallest. The predictive results of Covidex and Nextclade are reported in Table 3 for the original data. Overall, we can observe that the predictive performance for both of these methods is higher that the embedding methods we used including *k*-mers Vector, PSSM Vector, and Minimizer Vector. This is because both of these methods are already pre-trained on larger datasets and did not go through the typical training process that we used for the embedding methods, hence we reported their results separately.

**Table 2:** Accuracy results on 8220 (original) nucleotide sequences (without any error).

Embed. method	ML algo.	Acc.	Prec.	Recall	F1 weigh.	F1 macro	ROC-AUC	Train. runtime (s)
*k*-mers vector	SVM	**0.87**	**0.87**	**0.87**	**0.86**	**0.76**	**0.87**	7.43
	NB	0.03	0.05	0.03	0.02	0.05	0.55	0.09
	MLP	0.75	0.74	0.75	0.74	0.36	0.68	18.42
	KNN	0.73	0.73	0.73	0.71	0.48	0.71	2.04
	RF	0.82	0.85	0.82	0.80	0.67	0.78	2.17
	LR	0.86	0.85	0.86	0.85	0.70	0.84	8.67
	DT	0.67	0.67	0.67	0.66	0.42	0.71	0.27
PSSM vector	SVM	0.28	0.08	0.28	0.12	0.01	0.50	3.14
	NB	0.01	0.01	0.01	0.00	0.01	0.52	**0.03**
	MLP	0.34	0.27	0.34	0.26	0.06	0.53	17.31
	KNN	0.32	0.28	0.32	0.28	0.13	0.55	0.33
	RF	0.33	0.30	0.33	0.31	0.16	0.57	1.60
	LR	0.28	0.08	0.28	0.12	0.01	0.50	0.68
	DT	0.29	0.28	0.29	0.28	0.13	0.56	0.06
Minimizer vector	SVM	0.60	0.58	0.60	0.56	0.48	0.72	15.19
	NB	0.05	0.12	0.05	0.04	0.12	0.59	0.08
	MLP	0.57	0.52	0.57	0.53	0.30	0.64	26.32
	KNN	0.55	0.56	0.55	0.53	0.37	0.66	1.51
	RF	0.75	0.79	0.75	0.74	0.61	0.76	1.72
	LR	0.58	0.55	0.58	0.54	0.40	0.68	6.36
	DT	0.64	0.64	0.64	0.64	0.48	0.74	0.14

The best values are shown in bold.

**Table 3:** Accuracy results on 8220 (original) nucleotide sequences (without any error) using Covidex and Nextclade.

Method	Acc.	Prec.	Recall	F1 weigh.	F1 macro	ROC-AUC
Covidex	0.94	0.95	0.94	0.94	0.60	0.94
Nextclade	0.94	0.95	0.94	0.94	0.62	0.92

The standard deviation of 5 runs for the original data (without any errored sequences) is given in Table 4. Note that the accuracies (average values of 5 runs) for the same data are reported in Table 2 in the main paper.

**Table 4:** Standard deviation results on 8220 (original) nucleotide sequences (without any error).

Embed. method	ML algo.	Acc.	Prec.	Recall	F1 weigh.	F1 macro	ROC-AUC	Train. runtime (s)
*k*-mers vector	SVM	0.009337	0.008654	0.009337	0.009122	0.011612	0.011101	0.300614
	NB	0.003307	0.059135	0.003307	0.003323	0.002726	0.007099	0.017888
	MLP	0.012523	0.015917	0.012523	0.015363	0.028586	0.017015	3.836258
	KNN	0.009530	0.011883	0.009530	0.010848	0.022703	0.011329	0.036290
	RF	0.005541	0.006865	0.005541	0.006641	0.028757	0.014456	0.263474
	LR	0.005504	0.004573	0.005504	0.005868	0.017053	0.012580	0.867295
	DT	0.004102	0.002976	0.004102	0.003101	0.013446	0.010527	0.032883
PSSM vector	SVM	0.007223	0.003972	0.007223	0.005558	0.000217	0.000000	0.118888
	NB	0.002015	0.007148	0.002015	0.000983	0.004011	0.006194	0.008142
	MLP	0.005716	0.015879	0.005716	0.006257	0.009698	0.005917	2.603367
	KNN	0.009810	0.013064	0.009810	0.010757	0.023313	0.010837	0.028786
	RF	0.006478	0.008212	0.006478	0.007973	0.017069	0.007942	0.052490
	LR	0.007223	0.003972	0.007223	0.005558	0.000217	0.000000	0.025634
	DT	0.006273	0.009446	0.006273	0.007321	0.013302	0.007477	0.009866
Minimizer vector	SVM	0.008510	0.008449	0.009629	0.008932	0.015522	0.010247	0.281588
	NB	0.004464	0.060411	0.004464	0.006046	0.02021	0.007911	0.016351
	MLP	0.011599	0.007398	0.011599	0.009882	0.028691	0.015814	1.480781
	KNN	0.006601	0.009581	0.006601	0.007661	0.014181	0.005275	0.014744
	RF	0.004837	0.004967	0.004837	0.006467	0.034441	0.017324	0.044175
	LR	0.001902	0.005262	0.001902	0.002995	0.014008	0.004733	0.261828
	DT	0.010011	0.011210	0.010011	0.010262	0.024173	0.014445	0.011970

The results comparisons for DL-vs-Non DL methods are shown in Table 5. Since the DL methods show lower results than simple ML models, we only report the robustness results (in the next section) for ML models in this paper.

**Table 5:** Comparison of simple ML models with different DL methods on 8220 (original) nucleotide sequences (without any error).

Method	Embed. method	ML algo.	Acc.	Prec.	Recall	F1 weigh.	F1 macro	ROC- AUC	Train. runtime (s)
Non-DL methods	*k*-mers vector	SVM	**0.87**	**0.87**	**0.87**	**0.86**	**0.76**	**0.87**	7.43
		NB	0.03	0.05	0.03	0.02	0.05	0.55	0.09
		MLP	0.75	0.74	0.75	0.74	0.36	0.68	18.4
		KNN	0.73	0.73	0.73	0.71	0.48	0.71	2.04
		RF	0.82	0.85	0.82	0.8	0.67	0.78	2.17
		LR	0.86	0.85	0.86	0.85	0.70	0.84	8.67
		DT	0.67	0.67	0.67	0.66	0.42	0.71	0.27
		SVM	0.28	0.08	0.28	0.12	0.01	0.5	3.14
		NB	0.01	0.01	0.01	0.01	0.01	0.52	**0.03**
		MLP	0.34	0.27	0.34	0.26	0.06	0.53	17.3
	PSSM vector	KNN	0.32	0.28	0.32	0.28	0.13	0.55	0.33
		RF	0.33	0.3	0.33	0.31	0.16	0.57	1.6
		LR	0.28	0.08	0.28	0.12	0.01	0.5	0.68
		DT	0.29	0.28	0.29	0.28	0.13	0.56	0.06
		SVM	0.60	0.58	0.6	0.56	0.48	0.72	15.1
		NB	0.05	0.12	0.05	0.04	0.12	0.59	0.08
		MLP	0.57	0.52	0.57	0.53	0.3	0.64	26.3
	Minimizer vector	KNN	0.55	0.56	0.55	0.53	0.37	0.66	1.51
		RF	0.75	0.79	0.75	0.74	0.61	0.76	1.72
		LR	0.58	0.55	0.58	0.54	0.4	0.68	6.36
		DT	0.64	0.64	0.64	0.64	0.48	0.74	0.14
DL methods	LSTM	–	0.31	0.08	0.31	0.15	0.04	0.52	14894.06
	GRU	–	0.29	0.11	0.29	0.14	0.05	0.53	45890.74
	CNN	–	0.25	0.12	0.25	0.16	0.1	0.54	394775.6
Pre-trained	SeqVec	SVM	0.74	0.72	0.74	0.71	0.46	0.68	10.5
		NB	0.63	0.66	0.63	0.64	0.35	0.67	0.07
		MLP	0.75	0.72	0.75	0.72	0.44	0.70	19.7
		KNN	0.58	0.61	0.58	0.57	0.18	0.55	1.74
		RF	0.75	0.74	0.75	0.74	0.50	0.71	1.45
		LR	0.73	0.71	0.73	0.72	0.42	0.69	8.79
		DT	0.71	0.70	0.71	0.72	0.47	0.70	0.76

The best values are shown in bold.

### Robustness results

For the robustness results, we show the predictive performance of different ML models by first using the PBSIM-based noisy sequences and then show the performance of ML models for Illumina-based noisy examples.

For the PBSIM-based sequences, we take the original 8220 (non-errored) sequence data for training the ML models and use the PBSIM-based (8220) errored sequences as the test set. The purpose of this experimental setting is to evaluate the performance of ML models on the errored sequences, which were unavailable during the training process. In this experimental setting, we show the results for depth 5 and depth 10 (i.e., perturbation budgets) based errored sequences (in the test set) in Table 6. The robustness results for Covidex and Nextclade are reported in Table 7. Here, we can observe that although Covidex achieves higher predictive performance for noise data with depth 5, it completely fails to predict even a single sequence’s lineage correctly for depth 10 data. Moreover, Nextclade failed for both depth 5 and depth 10 data completely. This could happen due to the fact that these methods considered the errored mutations as some original mutations which were not present during their training process, hence they simply predicted lineage “B” for all of the noisy sequences (where the accuracy is 0), which means that these sequences could be any sub-category of a more generic “B” lineage. This behavior shows that these two methods do not generalize well to noisy sequences. Similarly, the robustness results of PBSIM-based errored sequences (with depth 15 and 20) are shown in Table 8. The robustness results of Covidex and Nextclade for depth 15 and 20 datasets are reported in Table 9. We can again observe that these two methods failed completely and only gave “B” as the predicted label. Hence, we can conclude that these methods cannot generalize well to the noisy data generated using the PBSIM simulator.

**Table 6:** Robustness results on PBSIM data with 5 and 10 as read depth.

Embed. method	ML algo.	Depth: 5							Depth: 10						
		Acc.	Prec.	Recall	F1 weigh.	F1 macro	ROC-AUC	Train. runtime (s)	Acc.	Prec.	Recall	F1 weigh.	F1 macro	ROC-AUC	Train. runtime (s)
*k*-mers vector	SVM	0.01	0.00	0.01	0.00	0.00	0.502	16.48	0.01	0.00	0.01	0.00	0.00	0.500	16.88
	NB	0.00	0.00	0.00	0.00	0.00	0.501	0.68	0.00	0.00	0.00	0.00	0.00	0.501	0.71
	MLP	0.282	0.083	0.285	0.123	0.01	0.505	23.65	0.02	0.00	0.02	0.00	0.00	0.507	16.86
	KNN	0.285	0.081	0.283	0.121	0.01	0.504	1.68	0.28	0.08	0.28	0.12	0.01	0.505	1.78
	RF	**0.289**	**0.085**	**0.289**	**0.124**	0.01	**0.509**	1.78	0.28	0.08	0.28	0.12	0.01	0.502	2.88
	LR	0.01	0.00	0.01	0.00	0.00	0.501	11.30	0.01	0.00	0.01	0.00	0.00	0.501	12.04
	DT	0.01	0.00	0.01	0.00	0.00	0.503	0.34	0.01	0.00	0.01	0.00	0.00	0.505	0.36
PSSM vector	SVM	0.27	0.07	0.27	0.11	0.01	0.504	8.14	0.30	0.09	0.30	0.13	0.01	0.506	8.32
	NB	0.27	0.07	0.27	0.11	0.01	0.501	0.34	0.30	0.09	0.30	0.13	0.01	0.508	0.36
	MLP	0.27	0.07	0.27	0.11	0.01	0.506	7.47	0.30	0.09	0.30	0.13	0.01	0.503	7.90
	KNN	0.27	0.07	0.27	0.11	0.01	0.502	0.51	0.01	0.05	0.01	0.00	0.00	0.502	0.52
	RF	0.27	0.07	0.27	0.11	0.01	0.507	1.17	0.302	0.096	0.302	0.130	0.012	0.505	0.98
	LR	0.27	0.07	0.27	0.11	0.01	0.503	3.76	0.301	0.095	0.301	0.131	0.016	0.501	3.62
	DT	0.27	0.07	0.27	0.11	0.01	0.501	**0.02**	**0.304**	**0.099**	**0.304**	**0.136**	**0.017**	**0.509**	**0.02**
Minimizer vector	SVM	0.27	0.07	0.26	0.11	0.01	0.506	5.22	0.27	0.08	0.27	0.12	0.01	0.501	4.91
	NB	0.26	0.07	0.27	0.11	0.265	0.502	0.43	0.27	0.08	0.27	0.12	0.01	0.504	0.34
	MLP	0.26	0.07	0.26	0.11	0.261	0.504	1.63	0.27	0.08	0.27	0.12	0.01	0.506	1.92
	KNN	0.26	0.07	0.26	0.11	0.263	0.506	0.62	0.08	0.01	0.08	0.01	0.00	0.503	0.69
	RF	0.26	0.07	0.26	0.11	**0.268**	0.501	0.67	0.27	0.08	0.27	0.12	0.01	0.502	0.77
	LR	0.26	0.07	0.26	0.11	0.267	0.502	0.69	0.27	0.08	0.27	0.12	0.01	0.504	0.67
	DT	0.26	0.07	0.26	0.11	0.266	0.505	0.17	0.27	0.08	0.27	0.12	0.01	0.501	0.26

The best values are shown in bold.

**Table 7:** Robustness results on PBSIM data with 5 and 10 as read depth using Covidex and Nextclade.

Method	Depth: 5						Depth: 10					
	Acc.	Prec.	Recall	F1 weigh.	F1 macro	ROC-AUC	Acc.	Prec.	Recall	F1 weigh.	F1 macro	ROC-AUC
Covidex	0.78	0.80	0.78	0.77	0.38	0.84	0.0	0.0	0.0	0.0	0.0	0.5
Nextclade	0.0	0.0	0.0	0.0	0.0	0.5	0.0	0.0	0.0	0.0	0.0	0.5

**Table 8:** Robustness results on PBSIM data with 15 and 20 as read depth.

Embed. method	ML algo.	Depth: 15							Depth: 20						
		Acc.	Prec.	Recall	F1 weigh.	F1 macro	ROC-AUC	Train. runtime (s)	Acc.	Prec.	Recall	F1 weigh.	F1 macro	ROC-AUC	Train. runtime (s)
*k*-mers vector	SVM	0.01	0.00	0.01	0.00	0.00	0.50	11.19	0.01	0.00	0.01	0.00	0.00	0.50	11.17
	NB	0.00	0.00	0.00	0.00	0.00	0.50	0.81	0.00	0.00	0.00	0.00	0.00	0.50	0.70
	MLP	0.00	0.00	0.00	0.00	0.00	0.50	21.66	0.01	0.00	0.01	0.00	0.00	0.50	20.82
	KNN	0.28	0.08	0.28	0.12	0.01	0.50	2.32	0.28	0.08	0.28	0.12	0.01	0.50	2.24
	RF	0.28	0.08	0.28	0.12	0.01	0.50	2.48	0.28	0.08	0.28	0.12	0.01	0.50	2.42
	LR	0.01	0.00	0.01	0.00	0.00	0.50	11.89	0.01	0.00	0.01	0.00	0.00	0.50	11.77
	DT	0.01	0.00	0.01	0.00	0.00	0.50	0.31	0.01	0.00	0.01	0.00	0.00	0.50	0.31
PSSM vector	SVM	0.28	0.08	0.28	0.12	0.01	0.50	9.97	0.28	0.08	0.28	0.12	0.01	0.50	9.54
	NB	0.01	0.00	0.01	0.00	0.00	0.50	0.15	0.01	0.00	0.01	0.00	0.00	0.50	0.16
	MLP	0.01	0.00	0.01	0.00	0.00	0.50	15.63	0.01	0.00	0.01	0.00	0.00	0.50	19.20
	KNN	0.01	0.00	0.01	0.00	0.00	0.50	2.21	0.01	0.00	0.01	0.00	0.00	0.50	2.18
	RF	0.00	0.00	0.00	0.00	0.00	0.50	2.03	0.00	0.00	0.00	0.00	0.00	0.50	1.94
	LR	0.28	0.08	0.28	0.12	0.01	0.50	1.13	0.28	0.08	0.28	0.12	0.01	0.50	1.11
	DT	0.00	0.00	0.00	0.00	0.00	0.50	0.09	0.00	0.00	0.00	0.00	0.00	0.50	0.08
Minimizer vector	SVM	0.01	0.01	0.01	0.00	0.01	0.50	15.47	0.01	0.01	0.01	0.00	0.01	0.50	17.54
	NB	0.00	0.00	0.00	0.00	0.00	0.50	0.77	0.00	0.00	0.00	0.00	0.00	0.50	0.84
	MLP	0.05	0.00	0.05	0.00	0.00	0.50	24.19	0.05	0.00	0.05	0.00	0.00	0.50	34.86
	KNN	0.05	0.00	0.05	0.00	0.00	0.50	2.50	0.05	0.00	0.05	0.00	0.00	0.50	1.93
	RF	0.09	0.01	0.09	0.01	0.00	0.50	2.39	0.28	0.08	0.28	0.12	0.01	0.50	2.25
	LR	0.00	0.00	0.00	0.00	0.00	0.50	9.45	0.00	0.00	0.00	0.00	0.00	0.50	10.11
	DT	0.07	0.01	0.07	0.01	0.00	0.50	0.20	0.07	0.01	0.07	0.01	0.00	0.50	0.19

**Table 9:** Robustness results on PBSIM data with 15 and 20 as read depth using Covidex and Nextclade.

Method	Depth: 15						Depth: 20					
	Acc.	Prec.	Recall	F1 weigh.	F1 macro	ROC–AUC	Acc.	Prec.	Recall	F1 weigh.	F1 macro	ROC–AUC
Covidex	0.0	0.0	0.0	0.0	0.0	0.5	0.0	0.0	0.0	0.0	0.0	0.5
Nextclade	0.0	0.0	0.0	0.0	0.0	0.5	0.0	0.0	0.0	0.0	0.0	0.5

For the Illumina-based sequences, we take the original 8220 (non-errored) sequence data for training the ML models and use the Illumina-based errored 8220 sequences as the test set. In this experimental setting, we show the results for sequences simulated using a different number of short reads (in the test set). The results for 5000 short reads and 10, 000 short reads (i.e., perturbation budget) based errored sequences are shown in Table 10. The robustness results for the same data using Covidex and Nextclade are reported in Table 11. Opposite to the results for the PBSIM simulator, we can observe that both Covidex and Nextclade show higher robustness results compared to the embedding methods. Similarly, the robustness results with Illumina-based errored sequences having the number of short reads as 15, 000 and 20, 000 are shown in Table 12. Moreover, the robustness results using Covidex and Nextclade on the same datasets are reported in Table 13. Other than Macro F1, both Covidex and Nextclade outperforms the embedding methods for all other evaluation metrics, hence showing better generalizability over the noisy sequences.

**Table 10:** Robustness results on illumina-based errored sequences with 5000 and 10, 000 short reads used in the simulation process.

Embed. method	ML algo.	# of short reads: 5000							# of short reads: 10,000						
		Acc.	Prec.	Recall	F1 weigh.	F1 macro	ROC-AUC	Train. runtime (s)	Acc.	Prec.	Recall	F1 weigh.	F1 macro	ROC-AUC	Train. runtime (s)
*k*-mers vector	SVM	0.68	0.66	0.68	0.66	0.49	0.73	6.75	0.732	0.72	0.71	0.722	0.55	0.76	10.76
	NB	0.69	0.73	0.69	0.71	0.571	**0.80**	0.31	0.72	0.72	0.72	0.721	0.53	**0.77**	0.32
	MLP	0.68	0.65	0.68	0.66	0.34	0.66	75.93	0.68	0.65	0.68	0.66	0.32	0.65	27.84
	KNN	**0.73**	**0.73**	**0.73**	**0.72**	**0.574**	0.76	0.75	0.731	0.72	**0.733**	**0.727**	**0.56**	0.76	0.68
	RF	0.72	0.72	0.72	0.70	0.51	0.72	2.44	**0.738**	**0.73**	0.731	0.71	0.55	0.74	2.43
	LR	0.72	0.70	0.72	0.70	0.52	0.74	6.71	0.72	0.71	0.72	0.71	0.54	0.75	6.69
	DT	0.51	0.53	0.51	0.52	0.32	0.66	0.24	0.56	0.56	0.56	0.56	0.41	0.70	0.21
PSSM vector	SVM	0.27	0.07	0.27	0.12	0.01	0.50	8.20	0.28	0.08	0.28	0.12	0.01	0.50	9.64
	NB	0.01	0.00	0.01	0.00	0.01	0.51	0.39	0.02	0.01	0.02	0.01	0.03	0.52	0.25
	MLP	0.32	0.22	0.32	0.24	0.06	0.52	10.30	0.34	0.25	0.34	0.26	0.08	0.53	12.72
	KNN	0.26	0.21	0.26	0.22	0.06	0.52	1.10	0.29	0.26	0.29	0.25	0.09	0.54	0.70
	RF	0.30	0.24	0.30	0.25	0.08	0.52	2.17	0.32	0.25	0.32	0.27	0.08	0.53	1.92
	LR	0.27	0.07	0.27	0.12	0.01	0.50	3.92	0.28	0.08	0.28	0.12	0.01	0.50	3.26
	DT	0.30	0.24	0.30	0.25	0.07	0.52	**0.121**	0.32	0.25	0.32	0.26	0.08	0.53	**0.07**
Minimizer vector	SVM	0.52	0.47	0.52	0.46	0.30	0.64	11.75	0.54	0.50	0.54	0.49	0.34	0.66	7.45
	NB	0.05	0.27	0.05	0.04	0.09	0.63	0.20	0.07	0.37	0.07	0.08	0.14	0.64	0.19
	MLP	0.52	0.46	0.52	0.46	0.26	0.62	25.0	0.52	0.46	0.52	0.48	0.25	0.62	28.70
	KNN	0.55	0.55	0.55	0.53	0.39	0.67	0.52	0.57	0.57	0.57	0.56	0.47	0.70	0.56
	RF	0.65	0.67	0.65	0.63	0.46	0.70	1.75	0.68	0.69	0.68	0.66	0.56	0.74	1.60
	LR	0.51	0.46	0.51	0.46	0.28	0.63	2.91	0.53	0.49	0.53	0.48	0.34	0.65	2.90
	DT	0.47	0.47	0.47	0.47	0.31	0.65	0.128	0.54	0.54	0.54	0.54	0.42	0.70	0.10

The best values are shown in bold.

**Table 11:** Robustness results on Covidex and Nextclade using illumina-based errored sequences with 5000 and 10, 000 short reads used in the simulation process.

Method	# of short reads: 5000						# of short reads: 10,000					
	Acc.	Prec.	Recall	F1 weigh.	F1 macro	ROC-AUC	Acc.	Prec.	Recall	F1 weigh.	F1 macro	ROC-AUC
Covidex	0.94	0.95	0.94	0.94	0.60	0.94	0.78	0.80	0.78	0.78	0.38	0.84
Nextclade	0.76	0.78	0.76	0.77	0.35	0.81	0.77	0.79	0.77	0.78	0.36	0.82

**Table 12:** Robustness results on illumina-based errored sequences with 15,000 and 20,000 short reads used in the simulation process.

Embed. method	ML algo.	# of short reads: 15,000							# of short reads: 20,000						
		Acc.	Prec.	Recall	F1 weigh.	F1 macro	ROC-AUC	Train. runtime (s)	Acc.	Prec.	Recall	F1 weigh.	F1 macro	ROC-AUC	Train. runtime (s)
*k*-mers vector	SVM	0.68	0.69	0.68	0.67	0.44	0.72	12.41	0.68	0.69	0.68	0.67	0.44	0.72	11.60
	NB	0.00	0.00	0.00	0.00	0.03	0.52	0.95	0.00	0.00	0.00	0.00	0.03	0.52	0.65
	MLP	0.63	0.64	0.63	0.62	0.32	0.67	26.30	0.63	0.65	0.63	0.63	0.31	0.66	20.25
	KNN	0.51	0.50	0.51	0.45	0.13	0.56	2.50	0.51	0.50	0.51	0.45	0.13	0.56	2.29
	RF	**0.7**1	**0.73**	**0.71**	0.68	**0.51**	0.71	3.17	**0.71**	**0.72**	**0.71**	0.68	**0.49**	0.71	2.64
	LR	**0.71**	0.70	**0.71**	**0.69**	0.47	**0.73**	12.22	**0.71**	0.70	**0.71**	**0.69**	0.47	**0.73**	12.22
	DT	0.53	0.55	0.53	0.53	0.35	0.68	0.33	0.52	0.54	0.52	0.52	0.31	0.66	0.32
PSSM vector	SVM	0.28	0.08	0.28	0.12	0.01	0.50	9.79	0.28	0.08	0.28	0.12	0.01	0.50	9.59
	NB	0.00	0.00	0.00	0.00	0.00	0.50	0.18	0.00	0.00	0.00	0.00	0.00	0.50	0.22
	MLP	0.22	0.20	0.22	0.14	0.03	0.51	15.89	0.18	0.18	0.18	0.14	0.02	0.51	19.92
	KNN	0.17	0.22	0.17	0.14	0.02	0.51	1.98	0.17	0.22	0.17	0.14	0.02	0.51	2.28
	RF	0.12	0.17	0.12	0.12	0.03	0.51	1.76	0.13	0.18	0.13	0.13	0.03	0.51	2.00
	LR	0.28	0.08	0.28	0.12	0.01	0.50	1.01	0.28	0.08	0.28	0.12	0.01	0.50	1.03
	DT	0.13	0.19	0.13	0.13	0.03	0.51	**0.09**	0.12	0.18	0.12	0.12	0.03	0.51	**0.08**
Minimizer vector	SVM	0.52	0.53	0.52	0.48	0.31	0.65	19.16	0.52	0.54	0.52	0.48	0.32	0.65	18.63
	NB	0.02	0.07	0.02	0.01	0.06	0.55	0.95	0.02	0.07	0.02	0.01	0.06	0.55	0.77
	MLP	0.50	0.45	0.50	0.45	0.22	0.59	33.36	0.52	0.48	0.52	0.48	0.29	0.63	37.64
	KNN	0.37	0.35	0.37	0.31	0.10	0.55	2.42	0.37	0.35	0.37	0.31	0.10	0.55	2.34
	RF	0.64	0.68	0.64	0.60	0.46	0.69	2.21	0.64	0.67	0.64	0.60	0.45	0.69	2.44
	LR	0.51	0.53	0.51	0.46	0.30	0.64	9.14	0.51	0.53	0.51	0.46	0.30	0.64	9.38
	DT	0.51	0.53	0.51	0.51	0.35	0.68	0.19	0.51	0.53	0.51	0.51	0.36	0.68	0.19

The best values are shown in bold.

**Table 13:** Robustness results on Covidex and Nextclade using illumina-based errored sequences with 15,000 and 20,000 short reads used in the simulation process.

Method	# of short reads: 15,000						# of short reads: 20,000					
	Acc.	Prec.	Recall	F1 weigh.	F1 macro	ROC-AUC	Acc.	Prec.	Recall	F1 weigh.	F1 macro	ROC-AUC
Covidex	0.78	0.80	0.78	0.78	0.38	0.84	0.78	0.80	0.78	0.78	0.38	0.84
Nextclade	0.77	0.79	0.77	0.78	0.36	0.82	0.77	0.79	0.77	0.78	0.36	0.82

### PBSIM versus illumina results discussion

Third-generation sequencing technologies such as PacBio and Oxford Nanopore Technologies (ONT), being newer than traditional high-throughput NGS technologies (e.g., Illumina), offer longer reads, which are useful to efforts such as haplotype assembly^60,61^. The drawback with these technologies is that they have lower coverage and contain more errors—up to $15\%$ error rate as compared to the less than $1\%$ with state-of-the-art Illumina^62–65^. Therefore, it is not surprising that perturbing the coverage in the case of Pacbio (PBSIM) based experiment had a larger effect (see Table 6) on the predictive performance of ML models as compared to the Illumina (InSilicoSeq) based experiment (see Table 10). The sequences submitted to GISAID (https://www.gisaid.org/) database are almost exclusively from high-throughput technologies^16^. Hence we got more stable results on the original sequences (without adding any additional error) extracted from GISAID (see Table 2).

For the PBSIM-based errored sequences, we can observe that PSSM Vector outperforms the other two embedding methods (see Table 6), which means that a sliding window-based approach (using *k*-mers or *m*-mers) is not desirable while dealing with Pacbio errors. This could be due to the fact that the PSSM Vector representation captures more long-range information than the shorter (length *k*) sliding window. Similarly, for the Illumina-based sequences, we can observe the opposite behavior (see Table 10), where the sliding window-based approaches are better than the position weight matrix-based embedding. This could be because, in PSSM Vector, the order of nucleotides is not preserved in as much detail (because we just take the position weight matrix and make it a 1-D vector by flattening it). In the sliding window-based approach, we are able to preserve the order information, which results in better predictive performance (because of less loss of information in generating the numerical embedding). This comes at the cost of it being a higher dimensional representation, of course.

## Limitations

We used feature engineering-based embeddings along with some typical neural network models for the experiments in this paper. Using an exhaustive list of end-to-end neural network models (such as one proposed in^33^ for microRNA prediction) could improve the benchmark dataset’s accuracy and/or robustness. These models could also help us to understand the behavior of noisy simulations in more detail. Moreover, we use the Illumina-based data with 5000, 10, 000, 15, 000, and 20, 000 short reads only—we believe that using a larger number of reads may improve the performance of the underlying classifier. The same is true for PBSIM data, where we use only 5 and 10 as read depth.

## Conclusion

In this paper, we use two different ways to test the robustness of ML models in terms of sequence classification. We test the accuracy and robustness of ML models using different embedding methods and concluded that for different simulation tools, different embedding methods perform better than others, and there is no clear winner that consistently outperforms in all scenarios. One interesting future extension is to use other embedding methods from the literature and also apply deep learning models for the classification of sequences. Studying noise simulations on other viruses (e.g., Zika) is also an interesting future extension. We would also like to explore some advanced deep learning methods, such as transformers, to study the robustness in the future.

## Data Availability

All data generated or analyzed during this study are included in this published article (and its supplementary information files).
